# Manual lymphatic drainage reduces tissue sodium and self-reported pain in women with lipedema: a quantitative MRI study

**DOI:** 10.3389/fcell.2026.1808579

**Published:** 2026-10-07

**Authors:** E Nöll, A Henkel, M.E. Cornely, R Dinnendahl, E Breuer, S Nowak, T Hucho, J. A. Luetkens, C. C. Pieper

**Affiliations:** 1 Department of Diagnostic and Interventional Radiology, University of Bonn, University Hospital of Bonn, Bonn, Germany; 2 Institute for Basic Lymphological Research, LY.SEARCH gGmbH, Düsseldorf, Germany; 3 Department of Anesthesiology and Intensive Care Medicine, Translational Pain Research, Medical Faculty and University Hospital of Cologne, University of Cologne, Cologne, Germany; 4 TheraNova, Centre for Physical Therapy, Cologne, Germany; 5 Department of Anesthesiology and Intensive Care Medicine, Pain Center, University Hospital of Cologne, University of Cologne, Cologne, Germany

**Keywords:** lipedema, lipohyperplasia dolorosa, lymphedema, manual lymphatic drainage, quantitative MRI

## Abstract

**Background:**

Lipedema is a chronic adipose tissue disorder characterized by a disproportionate accumulation of painful subcutaneous fat on the extremities. Manual lymphatic drainage (MLD) is routinely used as therapeutic intervention, but its immediate physiological effects remain unclear. We evaluated the immediate impact of MLD on tissue composition in Lipedema using multiparametric Magnetic-resonance-imaging (MRI).

**Materials and Methods:**

Fifteen women with Lipedema (5 women each for stages I/II/III; median age 43.7 [20.4–68.8] years), thirteen with lower-limb lymphedema (3/5/5 women for stages I/II/III; median age 57.6 [18.6–70.4] years) underwent quantitative MRI of the legs including volumetry, sodium MRI, proton-densitiy fat-fraction (PDFF), T1, T2 and fat-suppressed T2 mapping before and after a standardized 1 h MLD session. Five normal controls (median age 41.9 [23.3–66.2] years) were examined only before MLD. Regions of interest in skin, subcutaneous adipose tissue (SAT), and muscle were analyzed. Pain was assessed by numeric rating scale (NRS). Wilcoxon tests and Spearman correlations were applied to test for differences pre- and post-MLD as well as for associations between measurements and clinical parameters (p < 0.05).

**Results:**

At baseline, women with Lipedema showed higher tissue sodium and water-sensitive MRI measures compared to controls (skin sodium: median 17.7 vs. 12.3 mmol/L, p < 0.001; skin T2: 56 vs. 37 ms, p = 0.03), though lower than lymphedema (skin sodium: 32.1 mmol/L, skin T2: 70 ms). Tissue sodium in women with Lipedema correlated with clinical stage (r = 0.775, p = 0.001) and pain intensity (r = 0.656, p = 0.01). After MLD, perceived pain intensity decreased (median NRS 2 → 0, p = 0.007) and edema-related MRI metrics including tissue sodium decreased in women with Lipedema (skin sodium: 17.7 → 14.8 mmol/L, p = 0.001; skin T2: 56 → 43 ms, p = 0.001). Parameter changes in the same direction after MLD were detected in women with Lymphedema (skin sodium: 32.1 → 27.1 mmol/L, p = 0.001; skin T2: 70 → 61 ms, p = 0.001).

**Conclusion:**

Manual lymphatic drainage produces immediate, quantifiable changes in treated tissue in women with lipedema, indicating mobilization of sodium-rich interstitial fluid.

## Introduction

Lipedema–sometimes also called Lipohyperplasia dolorosa (LiDo) – is a chronic adipose tissue disorder characterized by a disproportionate, bilateral accumulation of painful subcutaneous fat on the extremities sparing the trunk (Cornely, 2023; Cornely, 2014; Kruppa et al., 2026). Despite a suspected prevalence of up to 10% of women (Kruppa et al., 2020), objective diagnostic markers are scarce, contributing to delayed or missed diagnosis (Szolnoky et al., 2011; Vyas and Adnan, 2024; Nankam et al., 2022). Typical clinical features include mechanical hypersensitivity and/or pain in the affected extremities, easy bruising and refractoriness to weight loss efforts (Cornely, 2023; Kruppa et al., 2020; Vyas and Adnan, 2024; Faerber et al., 2024). The pathophysiology remains incompletely understood; hypotheses span hormonal and genetic influences, microvascular and lymphatic dysfunction, and extracellular matrix remodeling. As a result, no curative therapy exists (Faerber et al., 2024; Amato et al., 2024). Conservative management, often delivered as complete decongestive therapy (CDT), combines compression, exercise, skincare, and manual lymphatic drainage (MLD) (Faerber et al., 2024). Patients frequently report symptomatic relief such as reduced heaviness and pain-reduction. In contrast to treatment of lymphedema, objective, quantitative evidence of treatment effects of CDT or MLD in lipedema has been limited (Szolnoky et al., 2011; Atan and Bahar-Özdemir, 2021).

Advanced MRI techniques provide quantitative tissue characterization that can capture fluid and fat composition noninvasively. Lipedema does usually not cause any silhouette-changing oedema, but it is controversial whether subcutaneous interstitial oedema is present. Sodium (23Na) MRI demonstrated elevated tissue sodium in skin and subcutaneous adipose tissue (SAT) in women with Lipedema (Crescenzi et al., 2018; Crescenzi et al., 2020), suggesting altered interstitial electrolyte/fluid handling and possibly inflammation. In a pilot work, multimodal conservative therapy including MLD was associated with reduced tissue sodium in Lipedema over several weeks (Donahue et al., 2017). However, attribution of measured changes to specific parts of multimodal therapy over weeks is difficult. Furthermore, additional parameters sensitive for tissue fluid content derived from MR relaxometry measuring T1-and T2-relaxation times may also be helpful in quantifying treatment effects on tissue edema. In lymphedema, where there is ample evidence of excess interstitial fluid, T2-mapping is sensitive to edema and decreases after MLD (Donahue et al., 2017; Donahue et al., 2020).

To investigate immediate (acute) effects of a single standardized MLD session, the present study therefore employed a multiparametric MRI protocol including quantitative T1-and T2-mapping, mDIXON-based fat-quantification, and sodium MRI in women with Lipedema of the lower extremities. Women with lower-limb lymphedema were included as a positive control to test whether the employed MRI metrics are able to detect known MLD effects. We hypothesized that, if fluid is present in Lipedema tissues, quantitative MRI would reveal measurable reductions in edema-related markers such as shorter T2-times and lower sodium content after MLD. Additionally, we explored correlations of measured MRI parameters with clinical parameters such as subjectively reported pain-levels.

## Materials and methods

### Study design and participants

This prospective interventional study enrolled adult women with a clinical diagnosis of Lipedema or of lower-limb Lymphedema as a control condition presenting at the University Hospital Bonn who

- where diagnosed by a certified and experienced clinician (M.C., dermatologist with a lymphological focus and over 30 years of experience),
- underwent MLD as part of their current routine therapy and
- gave written informed consent to participate in the study provided.

The clinical diagnosis of Lipedema was made after a full medical history and physical examination when patients presented with: 1) bilateral, disproportional increase in leg volume not responding to exercise or dietary measures (without involvement of the feet), 2) pain on pressure or touch, spontaneous pain and a feeling of heaviness, 3) easy bruising of the legs, 4) negative Stemmer’s sign and 5) no pitting edema.

Patients with lymphedema had to fulfil the following criteria: 1) chronic swelling of a leg due to lymphatic insufficiency diagnosed with lymphatic imaging (MR-lymphangiography, lymphoscintigraphy or indocyanine fluorescence lymphangiography), 2) positive Stemmer’s sign, 3) pitting edema (in early stages).

Healthy participants had to be free from a history of leg swellings, surgeries on the legs or in the pelvis as well as kidney or heart diseases.

Patients and control subjects could in general not participate in the study during pregnancy and breastfeeding, or if they had other causes for edema (e.g., heart or kidney disease) or uncontrolled hypertension. Patients with implanted non-MR-compatible ferromagnetic intracorporeal devices such as cardiac pacemakers were also excluded. The study was approved by the local institutional review board (No. 237/22).

### Study procedure

Patient weight, height and waist circumference were measured and patients were asked to rate their current level of perceived pain in the legs using a numerical rating scale (NRS; 0: no pain, 10: worst imaginable pain) (Dinnendahl et al., 2024). All participants then underwent multiparametric MRI examination after laying flat for at least 10 min as detailed below. Immediately thereafter, standardized MLD was delivered for 60 min by a certified therapist (E.B.) following a proximal-to-distal protocol: bilateral cervical and axillary node stimulation, deep abdominal drainage, then lower-limb drainage (inguinal nodes, followed by rhythmic strokes/pumps along the limb—anterior, lateral, posterior—from thigh to foot). During this procedure patients remained primarily in a supine position. Thereafter, patients were asked to rate pain intensity levels again on a NRS and a second MRI scan was performed using the same protocol as before the MLD treatment. Participants were asked to refrain from drinking between the MRI examinations and were transported between MRI scanner and MLD therapy area by wheelchair to minimize activity between MRI examinations and therapy (Faerber et al., 2024).

### MRI acquisition

MRI targeted a matched axial slice at the proximal lower leg (calf), acquired pre-MLD and post-MLD with identical parameters. Two clinical MRI systems were used: a 3.0 T system (Achieva, Philips Healthcare, Best, Netherlands) with a dual-tuned 23Na/1H knee coil (Rapid Biomedical, Rimpar, Germany) for sodium MRI with co-registered axial multi-echo mDIXON for anatomy with a four-vial NaCl calibration phantom (10/20/30/40 mmol/L) to convert sodium signal to physiologic tissue sodium content (mmol/L) (Kopp et al., 2013); and a 1.5 T system (Ingenia, Philips Healthcare, Best, Netherlands) for quantitative mapping of T1-relaxation time as well as mapping of T2-relaxation times with and without fat-suppression employing 1H-relaxometry, and proton-density fat fraction (PDFF) mapping by multi-echo mDIXON fat quantification (Figure 1). Slice prescription on 1.5T matched the sodium slice using mDIXON references. Additionally morphological T1-weighted mDIXON scans of the whole legs with a field-of-view including both legs were acquired for 3D-volumetry. Detailed imaging parameters are given in Table 1.

**FIGURE 1:**
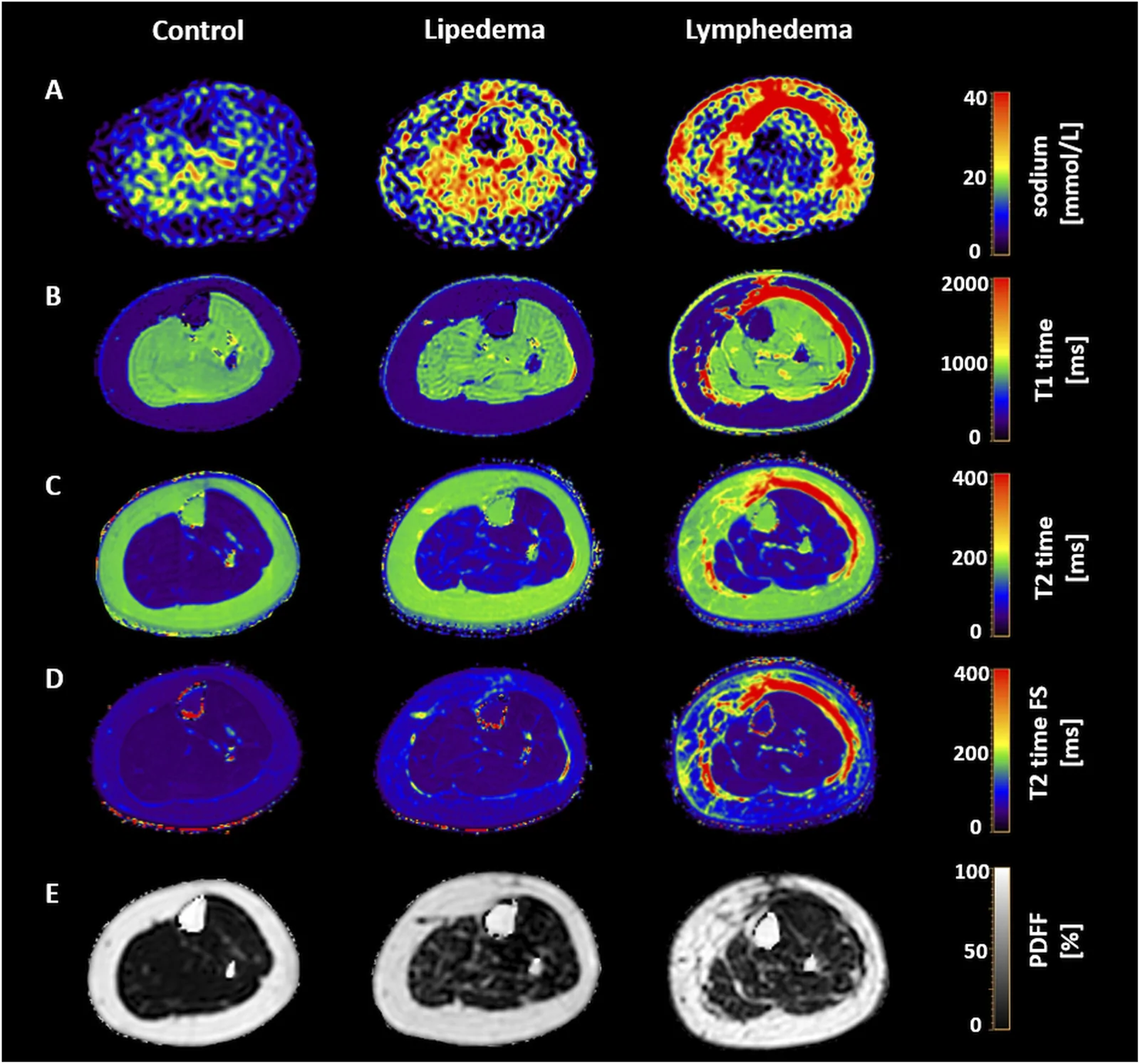
Example MRI scans of the lower leg **(A)** Sodium MRI **(B)** T1-mapping **(C)** T2-mapping **(D)** T2-mapping with fat-suppression **(E)** PDFF in a healthy woman, in a woman with Lipedema and a woman with Lymphedema.

**TABLE 1:** MRI parameters.

Parameter	Sodium scan (3T)	T1 mapping (1.5T)	T2 mapping (fatsat; 1.5T)	T2 mapping (non-fatsat; 1.5T)	mDIXON fatquant (1.5T)
Field-of-view (FOV)	192 × 192	450 × 274	450 × 274	450 × 274	450 × 285
Acquisition matrix	64 × 64	280 × 122	280 × 122	280 × 122	224 × 144
Voxel size acq (mm^2^) Rec (mm^2^)	3 × 3 3 × 3	1.62 × 2.26 1.1 × 1.1	1.62 × 2.26 1.1 × 1.1	1.62 × 2.26 1.1 × 1.1	2 × 2 1 × 1
Slice thickness	30 mm	12 mm	12 mm	12 mm	2 mm
TR	130 (ms)	3.1 (ms)	3,000	3,000	8.1 (ms)
TE	0.99 (ms)	1.32 (ms)	7.5 (ms)	7.5 (ms)	1.15 (ms)
Delta TE	​	​	​	​	1.1 (ms)
Number of echoes	​	6	32	32	6
Compressed SENSE/SENSE factor	​	CS 1.8	S 3	S 3	S 2
Fat sat	​	No	Yes	No	No
Profile order	​	Linear	Linear	Linear	​
TSE/TFE shot duration	​	234 (ms)	240 (ms)	240 (ms)	​
Bandwidth (Hertz/Pixel)	541.1	422.8	217.2	217.2	1771.5
Acquisition time	15 min	0:25 min	1:30 min	1:30 min	0:30 min

### Image analysis

Quantitative image analysis was performed by two blinded radiologists in consensus (C.C.P. with 14 years of experience, and A.H. with 5 years of experience) using a ROI-based approach in three compartments: skin, SAT, and muscle (excluding bone). ROIs were drawn on morphological mDIXON images and transferred to parametric maps (T1, T2, PDFF, sodium). Sodium images were calibrated against the phantom to yield mmol/L. Whole leg mDIXON imaging enabled automated tissue segmentation into a subcutaneous and a subfascial tissue compartment to estimate SAT and muscle volumes of the examined limbs, respectively, using a recently published artificial intelligence based workflow (Nowak et al., 2023). To ensure comparable results between MRI-scans, anatomical landmarks (i.e., the center of the femoral head and the ankles) were detected by the algorithm and used as the upper and lower margin for tissue volume calculation.

### Statistical analysis

All statistical analyses were performed using SPSS (version 22.0, IBM, Armonk, NY) and GraphPad (version 10.0.0 for Windows, GraphPad Software, Boston, Massachusetts). Data are given as median and range. Given a non-normal distributions (assessed by Q-Q-plots), Mann-Whitney tests were employed to compare baseline characteristics of patients with lipedema and lymphedema with control subjects. Wilcoxon signed-rank test was used to compare pre-vs. post-MLD values within groups. Spearman correlations were employed to assess associations between baseline imaging metrics and clinical variables (disease stage, NRS) as well as between NRS changes and tissue sodium changes. A multivariate linear regression was then used to explore independent imaging predictors of reported pain intensities (NRS). Significance was set at p < 0.05. The study was not powered or designed for inter-group comparison between patients with lipedema and lymphedema.

As an exploratory sensitivity analysis, baseline-adjusted linear regression models were used separately for the lipedema and lymphedema groups. For each MRI parameter, the post-MLD value was modeled with the corresponding pre-MLD value as covariate. These models were not used to compare lipedema and lymphedema directly, as the lymphedema cohort served as a positive control group and the study was not designed for inferential inter-group comparisons.

## Results

### Participant characteristics

15 women with Lipedema were included (five each with stage I, II and III, respectively) (median age 43.7 years [range 20.4–68.8]). 13 women with lymphedema (three, five, and five women for stages I, II, III, respectively) and a median age of 57.6 years (18.6–70.4) were included as a positive control group. 10/13 women (77%) suffered from secondary lymphedema due to gynecological tumor surgery and 3/13 women (23%) from primary lymphedema. In 12/13 cases (92.3%) the lymphedema was unilateral. Additionally, five healthy women (median age 41.9 years [range 23.3–66.2]) were included to determine baseline values before MLD. The healthy control group did not significantly differ in age distribution or WtHR from the patient groups with lipedema or lymphedema. BMI was slightly higher in the lipedema group compared to healthy controls with borderline significance and lower in the lymphedema group, without reaching statistical significance. See Table 2 for details.

**TABLE 2:** Patient characteristics.

Characteristic	Controls (n = 5)	Lipedema (n = 15)	p-value Lymphedema vs. Controls	Lymphedema (n = 13)	p-value Lipedema vs. Controls
Age (median, range; IQR)	41.9 (23.3–66.2; 38.5)	43.7 (20.4–68.8; 29.4)	0.965	57.6 (18.6–70.4; 22.2)	0.402
Female sex	5	15	​	13	​
Clinical diagnosis					
Lipedema Lymphedema	0 0	15 0	​	0 13	​
BMI (median, range; IQR)	30.1 (25.9–32.0; 5.2)	34.3 (23.0–41.1; 18.1)	0.05	23.1 (20.4–34.3; 9.4)	0.183
WtHR (median, range; IQR)	0.57 (0.5–0.67; 0.14)	0.64 (0.49–0.85; 0.14)	0.239	0.51 (0.43–0.68; 0.1)	0.301
Clinical stage					
Stage I Stage II Stage III	0 0 0	5 5 5	​	3 5 5	​
Involved side					
Right Left Both	0 0 0	0 0 15	​	5 7 1	​

IQR: interquartile range; BMI: body mass index; WtHR: waist-to-height ratio; waist to height ratio. P-values derived from Mann-Whitney Tests.

### Baseline characteristics of women with lipedema

Women with Lipedema reported baseline pain intensity levels with a median NRS of 2 (range 0–8) while normal controls and lymphedema patients reported pain intensities of 0 (0–1) and 2 (0–5).

At baseline, women with Lipedema showed significantly higher volumes of SAT compared to normal controls (25.3 vs. 17.2L, p = 0.02) while muscle volume was not significantly different (16.7 vs. 15.6L, p = 0.261). Tissue sodium was significantly higher in women with Lipedema than in healthy controls in the skin, SAT and muscle (17.7 vs. 12.3 mmol/L; 13.6 vs. 8.2 mmol/L and 23.1 vs. 15.2 mmol/L, respectively; all p < 0.001). T2-times were both significantly longer in women with Lipedema in the skin and muscle (56 vs. 37 ms, p = 0.03 and 61 vs. 42 ms, p = 0.03, respectively) while there was no difference in the SAT (167 vs. 160 ms, p = 0.353) as measurements in the SAT are dominated by fat signal. However, measuring T2-time with fat-suppression revealed a significantly longer T2-time also for the SAT in Lipedema compared to control subjects (68 vs. 55 ms, p = 0.001). T1-times were longer in Lipedema skin (755 vs. 441 ms, p = 0.01), but not for SAT and muscle (238 vs. 220 ms, p = 0.546 and 833 vs. 792 ms, p = 0.179). Conversely, total (including skin, SAT and muscle) and muscle (including only tissue below the muscle fascia) PDFF were significantly higher in women with Lipedema (52.2% vs. 40.0%, p = 0.02 and 12.2% vs. 6.4%, p < 0.001) and slightly higher also in the SAT (87.9% vs. 84.3%, p = 0.06). See Table 3 and Figure 2 for details.

**TABLE 3:** Measurements before and after MLD.

Parameter	Controls (n = 5)	Lipedema (n = 15)				Lymphedema (n = 13)			
​	​	Pre-MLD	Post-MLD	p-value	Baseline adjusted p-value	Pre-MLD	Post-MLD	p-value	Baseline adjusted p-value
NRS (0–10)	0 (0–1)	2 (0–8)	0 (0–7.5)	**0.007**	​	2 (0–5)	0 (0–4)	**0.039**	​
Tissue volume [L]									
SAT	17.2 (7.9–26.8)	25.3 (11.9–32.1)	24.9 (11.9–31.6)	0.132	0.196	13.2 (7.4–26.0)	13.2 (7.5–26.4)	0.727	0.994
Muscle	15.6 (12.6–20.9)	16.7 (13.7–20.3)	16.5 (13.4–20.1)	0.182	0.581	15.2 (12.3–21.2)	15.1 (12.2–21.3)	0.208	0.206
Tissue sodium [mmol/L]									
Skin	12.3 (9.4–14.1)	17.7 (13.1–29.6)	14.8 (10.9–19.9)	**0.001**	**<0.001**	32.1 (21.1–59.1)	27.1 (13.4–42.8)	**0.001**	**<0.001**
SAT	8.2 (7.6–10.3)	13.6 (10.9–16.4)	11.2 (6.8–13.6)	**0.001**	**<0.001**	36.1 (17.2–64.9)	33.3 (10.2–57.4)	**0.001**	**<0.001**
Muscle	15.2 (13.2–17.1)	23.1 (18.5–27.2)	20.3 (17.4–29.2)	**0.022**	**0.016**	32.1 (21.7–38.2)	34.2 (26.5–45.1)	**0.001**	**<0.001**
PDFF [%]									
Total	40.0 (24.2–46.0)	52.2 (40.5–60.4)	51.4 (41.0–60.0)	0.068	0.071	41.2 (33.1–54.3)	42.8 (33.5–55.1)	**0.003**	**0.005**
SAT	84.3 (83.2–86.4)	87.9 (80.9–90.5)	87.0 (80.5–91.5)	0.132	0.954	73.9 (47.1–91.8)	74.9 (48.9–92.2)	**0.08**	**0.109**
Muscle	6.4 (2.0–7.6)	12.2 (8.1–21.2)	12.0 (7.9–21.0)	0.111	0.213	13.1 (5.9–18.8)	13.5 (5.4–18.5)	0.582	0.920
T2 time [ms]									
Skin	37 (30–40)	56 (28–82)	43 (22–68)	**0.001**	**<0.001**	70 (64–101)	61 (53–81)	**0.001**	**<0.001**
SAT	160 (157–163)	167 (158–176)	168 (160–177)	0.154	0.094	213 (159–349)	198 (158–345)	**0.016**	**0.007**
Muscle	42 (38–54)	61 (47–82)	56 (47–79)	0.065	0.090	63 (46–96)	63 (50–83)	0.972	0.479
T2+FS time [ms]									
SAT	55 (50–58)	68 (56–88)	62 (51–82)	**0.001**	**<0.001**	221 (57–401)	200 (51–384)	**0.001**	**<0.001**
Muscle	42 (41–47)	52 (44–63)	49 (44–64)	0.200	0.267	54 (43–92)	55 (45–72)	0.480	0.084
T1 time [ms]									
Skin	441 (435–452)	755 (525–868)	705 (451–861)	**0.007**	**0.028**	1,063 (646–1,775)	942 (552–1,830)	**0.006**	**0.006**
SAT	220 (215–234)	238 (212–279)	241 (216–292)	0.116	0.102	512 (242–1,437)	545 (265–1,308)	0.294	0.112
Muscle	792 (742–848)	833 (799–914)	831 (795–915)	0.235	0.060	870 (805–948)	867 (822–910)	0.675	0.373

NRS: numeric rating scale for pain; PDFF: proton density fat fraction; FS: fat suppression; values given in median and range; p-values derived from Wilcoxon tests. Values < 0.05 (given in bold) are considered statistically significant. Baseline-adjusted p-values are derived from within-group linear regression models with change from baseline as dependent variable and the corresponding centered baseline value as covariate.

**FIGURE 2:**
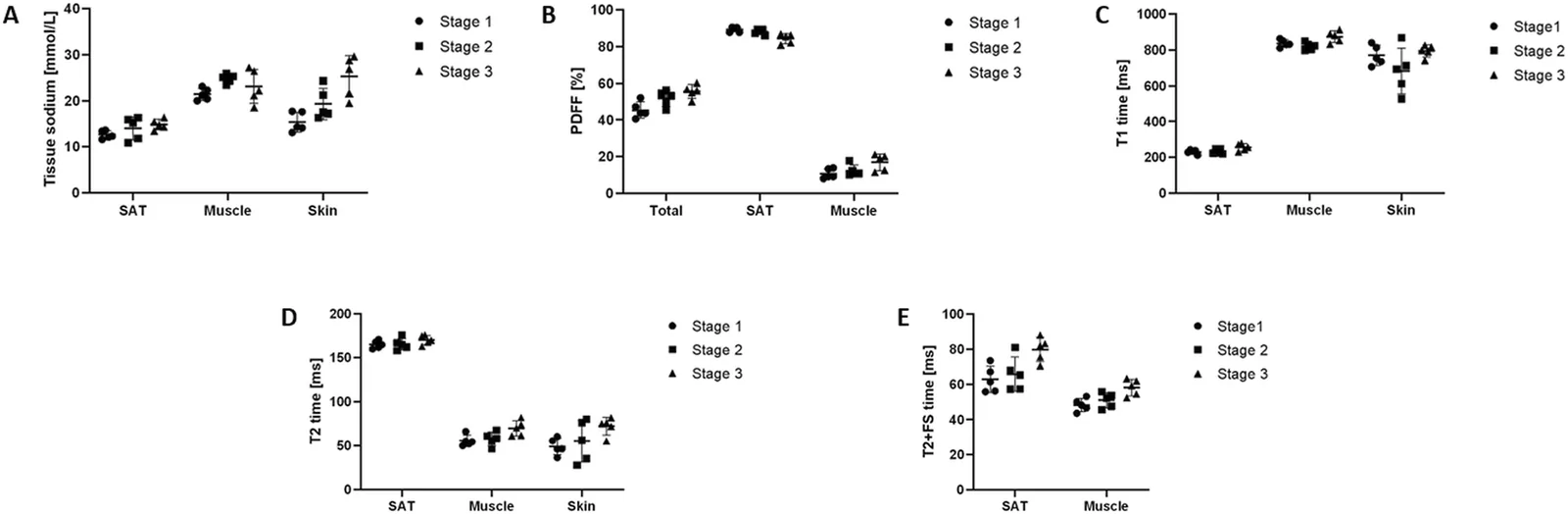
Baseline MRI parameter measurements in women with Lipedema **(A)** Tissue sodium **(B)** proton-density fat-fraction (PDFF) **(C)** T1-time **(D)** T2-time **(E)** T2-time with fat-suppression.

In women with Lipedema, baseline NRS values correlated with skin sodium (r = 0.656, p = 0.01), SAT T2-FS (r = 0.594, p = 0.022) and total PDFF (r = 0.586, p = 0.024). In multivariate analysis, skin sodium independently predicted baseline NRS values (p = 0.001). Additionally also clinical Lipedema stage correlated with skin sodium (r = 0.775, p = 0.001), SAT T2-FS (r = 0.699, p = 0.006), and total PDFF (r = 0.756, p = 0.002). See Table 4 and Figure 3 for details.

**TABLE 4:** Correlation of clinical and imaging parameters at baseline.

MRI parameter	Lipedema				Lymphedema			
​	Clinical staging		NRS pre MLD		Clinical staging		NRS pre MLD	
​	r	p	r	p	r	p	r	p
Tissue volume								
SAT	0.699	**0.006**	0.524	**0.047**	0.328	0.273	0.155	0.611
Muscle	0.199	0.482	−0.064	0.820	0.275	0.361	−0.236	0.433
Tissue sodium								
Skin	0.775	**0.001**	0.656	**0.01**	0.891	**<0.001**	0.610	**0.03**
SAT	0.511	0.056	0.314	0.253	0.938	**<0.001**	0.446	0.128
Muscle	0.283	0.317	0.205	0.461	0.721	**0.007**	0.201	0.505
PDFF								
Total	0.756	**0.002**	0.586	**0.024**	−0.138	0.651	−0.177	0.881
SAT	−0.794	**0.001**	−0.545	**0.038**	−0.609	**0.031**	−0.105	0.559
Muscle	0.586	**0.026**	0.215	0.439	0.293	0.330	0.047	0.732
T2 time								
Skin	0.491	0.070	0.227	0.414	0.586	**0.039**	0.157	0.604
SAT	0.370	0.178	0.200	0.470	0.927	**<0.001**	0.422	0.150
Muscle	0.680	**0.008**	0.363	0.184	0.065	0.835	−0.104	0.734
T2+FS time								
SAT	0.699	**0.006**	0.594	**0.022**	0.733	**0.006**	0.210	0.487
Muscle	0.699	**0.006**	0.425	0.116	0.287	0.340	0.201	0.505
T1 time								
Skin	0.151	0.608	−0.187	0.503	0.205	0.500	−0.315	0.291
SAT	0.529	**0.049**	0.337	0.219	0.850	**<0.001**	0.315	0.291
Muscle	0.454	0.098	0.480	0.073	0.234	0.440	−0.207	0.493

p-values derived from Spearman correlation analyses. Values < 0.05 (given in bold) are considered statistically significant.

**FIGURE 3:**
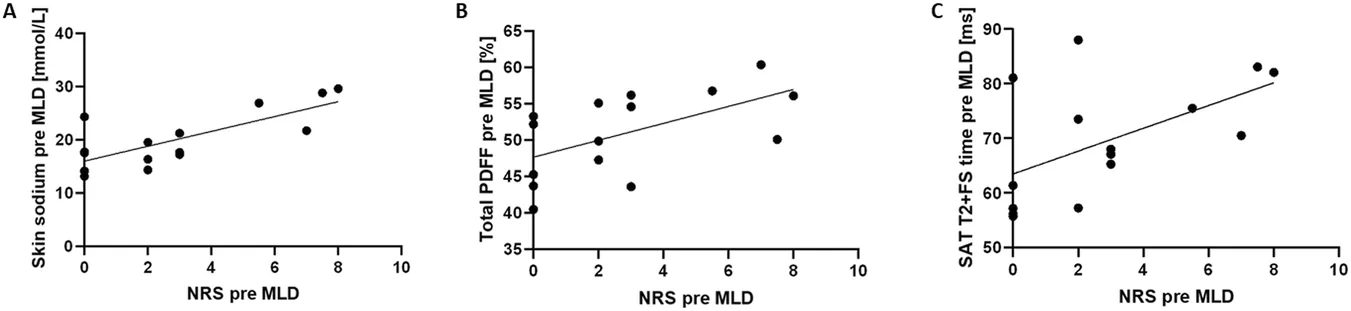
Correlation analyses between NRS values and MRI parameters in lipedema **(A)** Skin sodium **(B)** total proton-density fat-fraction (PDFF) **(C)** T2-time with fat-suppression.

### Baseline characteristics of women with lymphedema

In contrast, lymphedema patients showed significantly lower volumes of SAT compared to normal controls (13.2 vs. 17.2L, p = 0.03) while muscle volume was not significantly different (15.2 vs. 15.6L, p = 0.652). Otherwise, lymphedema patients showed differences in the same direction as women with Lipedema but to a larger extent: Tissue sodium was significantly higher in lymphedema patients than in healthy controls in the skin, SAT and muscle (32.1 vs. 12.3 mmol/L; 36.1 vs. 8.2 mmol/L and 32.1 vs. 15.2 mmol/L, respectively; all p < 0.001). T2-times were both significantly longer in the skin, SAT and muscle (70 vs. 37 ms, p = 0.001, 213 vs. 160 ms, p = 0.03 and 63 vs. 42 ms, p = 0.02, respectively). T2-time with fat-suppression showed these differences even more pronounced with significantly longer T2-time for SAT in lymphedema compared to control subjects (221 vs. 55 ms, p < 0.001). T1-times were also longer in lymphedema skin and SAT (1,063 vs. 441 ms, p < 0.001 and 512 vs. 220 ms, p = 0.01), but not for muscle (870 vs. 792 ms, p = 0.006). Total PDFF did not differ between lymphedema patients and controls (41.2% vs. 40.0%, p = 0.298), as it was significantly lower in the SAT (73.9% vs. 84.3%, p = 0.02), but significantly higher in muscle (13.1% vs. 6.4%, p < 0.001). See Table 3 and Figure 4 for details.

**FIGURE 4:**
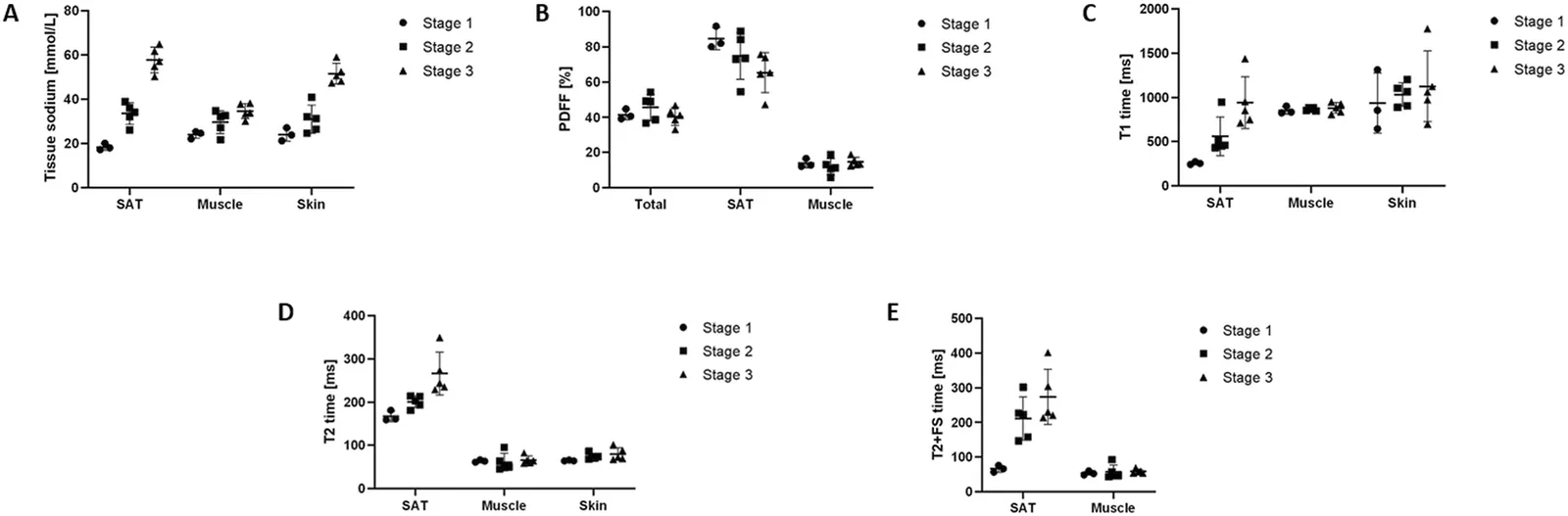
Baseline MRI parameter measurements in women with Lymphedema for skin, SAT and muscle shown for stages 1, 2 and 3 **(A)** Tissue sodium **(B)** proton-density fat-fraction (PDFF) **(C)** T1-time **(D)** T2-time **(E)** T2-time with fat-suppression.

In lymphedema, baseline NRS correlated with skin sodium (r = 0.610, p = 0.03) and clinical stage correlated with skin sodium (r = 0.891, p < 0.001), SAT sodium (r = 0.938, p < 0.001), muscle sodium (r = 0.721, p = 0.007), skin T2 (r = 0.586, p = 0.039) and SAT T2 (r = 0.927, p < 0.001), as well as SAT T2-FS (r = 0.733, p = 0.006) (Table 4; Figure 5).

**FIGURE 5:**
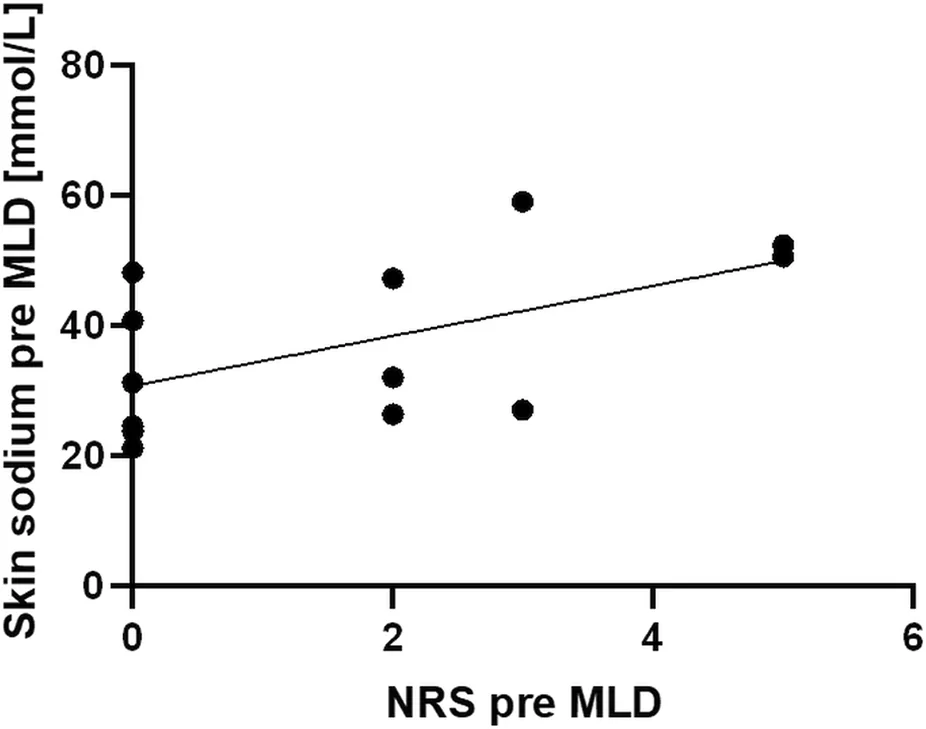
Correlation analysis between pain intensity values and skin sodium in lymphedema.

### Effects of MLD

In women with Lipedema, NRS-values decreased significantly after MLD (NRS 2 → 0, p = 0.007) (Figure 6). There was a tendency of lower SAT volume, but without reaching statistical significance (25.3 → 24.9 L, p = 0.132). Sodium levels consistently fell significantly in the skin (17.7 → 14.8 mmol/L, p = 0.001), SAT (13.6 → 11.2 mmol/L, p = 0.001) and muscle (23.1 → 20.3 mmol/L, p = 0.022). There was a moderate correlation between changes in NRS and tissue sodium in SAT (r = 0.52, p = 0.048), but no significant correlation for muscle or skin. T2 times were shortened significantly in the skin (56 → 43 ms, p = 0.001), but not in SAT and muscle, possibly again due to the dominant fat signal especially in the SAT. Accordingly, SAT T2 times were significantly shorter after MLD when adding fat-suppression (68 → 62 ms, p = 0.001). T1 time in the skin was shorter after MLD (755 → 705 ms, p = 0.007), but did not show significant changes in SAT or muscle. PDFF did not change significantly after MLD (total PDFF: 52.2% to 51.4%, p = 0.068; SAT PDFF: 87.9% to 87.0%, p = 0.132). See Table 3 and Figures 7, 8.

**FIGURE 6:**
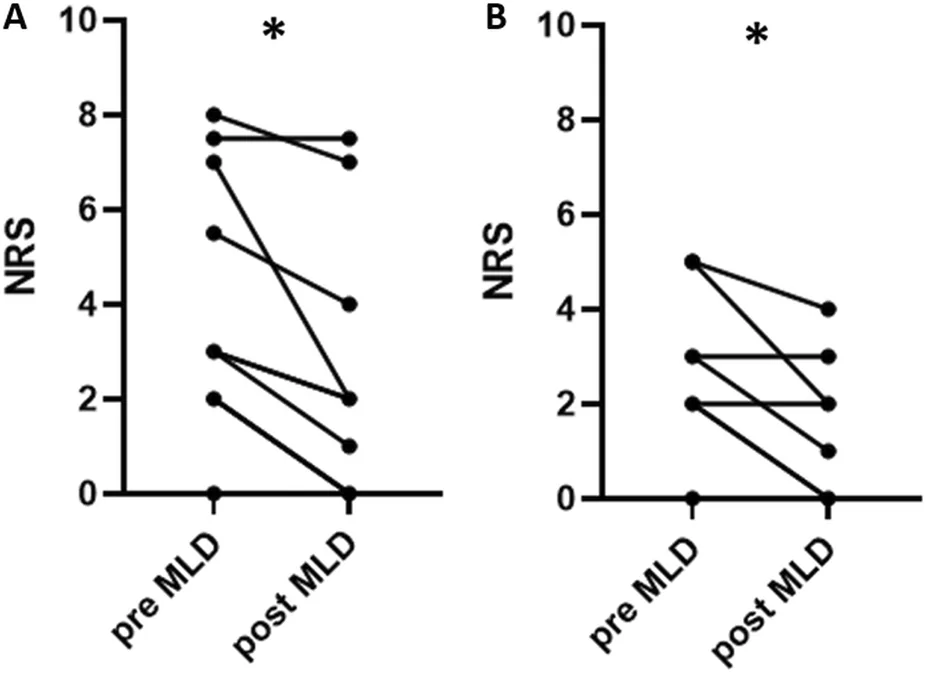
Changes in patient self-reported pain intensity levels before and after MLD. Pain intensity assessed by numerical rating scale (NRS; 0: no pain; 10: worst imaginable pain) in patients with **(A)** Lipedema and **(B)** Lymphedema. n. s nonsignificant change, * significant change.

**FIGURE 7:**
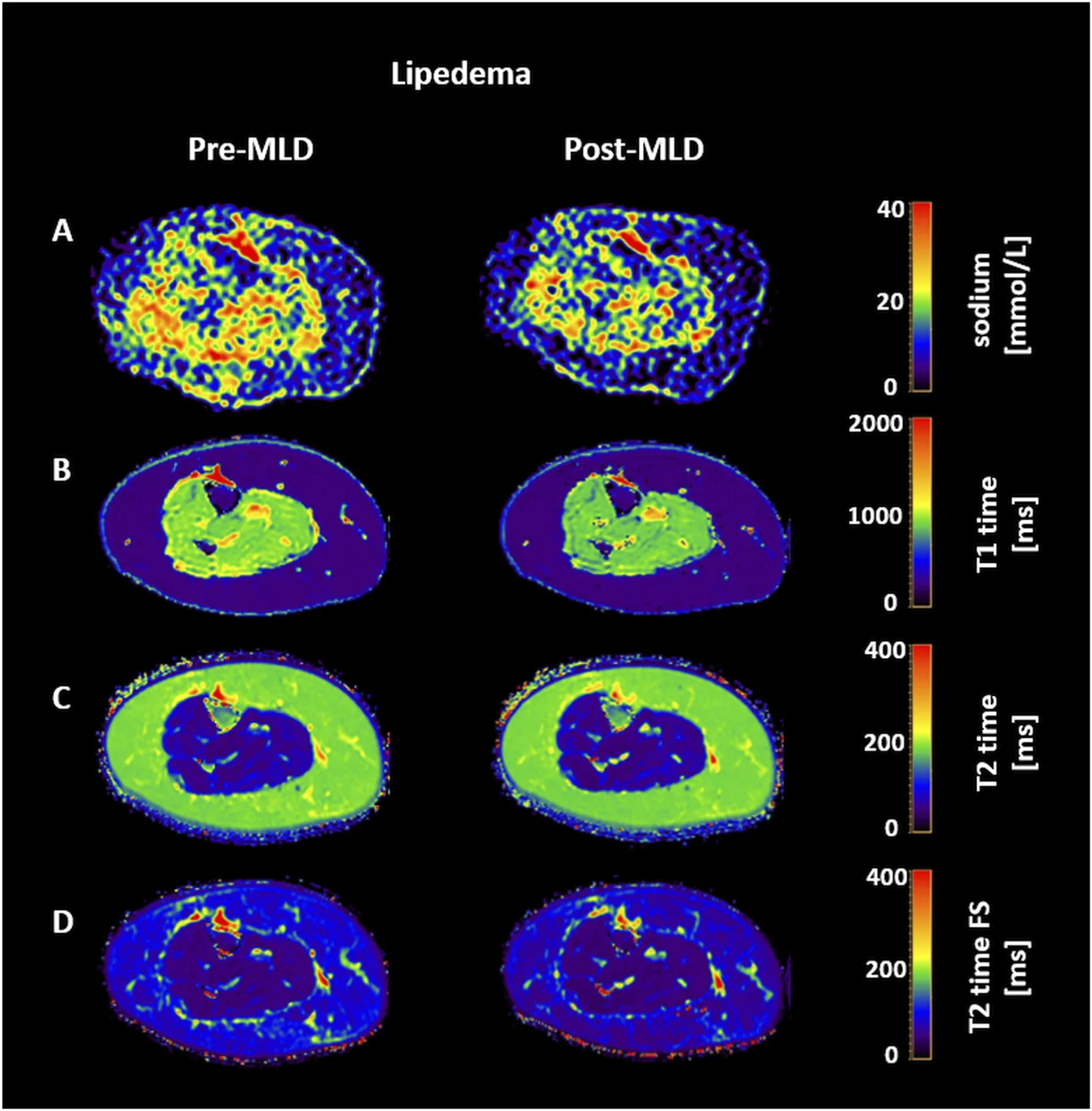
Example MRI scans of a 54-year-old woman with Lipedema **(A)** Sodium MRI **(B)** T1-mapping **(C)** T2-mapping **(D)** T2-mapping with fat-suppression.

**FIGURE 8:**
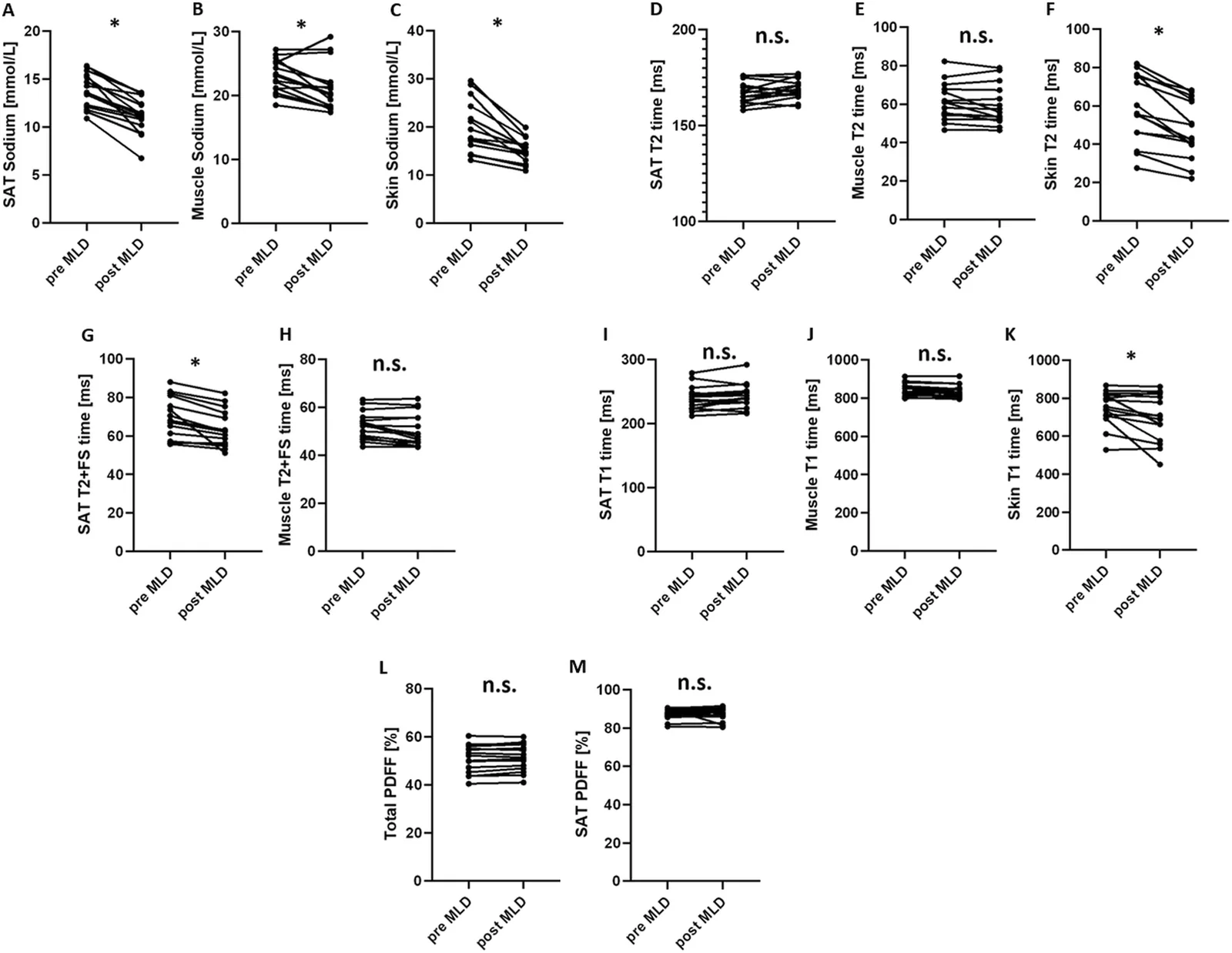
Changes after MLD in Lipedema. MRI parameters before and after MLD in patients with lipedema **(A)** SAT sodium **(B)** muscle sodium **(C)** skin sodium **(D)** SAT T2 time **(E)** muscle T2 time **(F)** skin T2 time **(G)** SAT T2 time with fat-suppression **(H)** muscle T2 time with fat-suppression **(I)** SAT T1 time **(J)** muscle T1 time **(K)** skin T1 time **(L)** SAT PDFF **(M)** total PDFF. n. s nonsignificant change, * significant change.

In patients with lymphedema, reported NRS-values decreased after MLD (2 → 0, p = 0.039) (Figure 6). SAT and muscle volume was not significantly affected by MLD in lymphedema. Otherwise, lymphedema patients showed parameter changes mostly in the same direction as in women with Lipedema patients post-MLD, but with larger absolute value changes: Sodium decreased significantly in the skin (32.1 → 27.1 mmol/L, p = 0.001) and SAT (36.1 → 33.3 mmol/L, p = 0.001); however, muscle sodium increased (32.1 → 34.2 mmol/L, p = 0.001). There was no significant correlation between NRS-changes and tissue sodium changes in patients with lymphedema. T2 times also decreased in the skin (70 → 61 ms, p = 0.001) and SAT (213 → 198 ms, p = 0.016), but did not change in muscle. Again, the decrease in T2 times was more pronounced in the SAT when applying a fat-suppressed technique (221 → 200 ms, p = 0.001). T1 time was shorter after MLD in the skin (1,063 → 942 ms, p = 0.006), but did not show significant changes in SAT or muscle. PDFF showed higher fat fraction post-MLD in lymphedema with total PDFF (41.2 → 42.8%, p = 0.003) and SAT PDFF (73.9 → 74.9%, p = 0.08). See Table 3 and Figures 9, 10.

**FIGURE 9:**
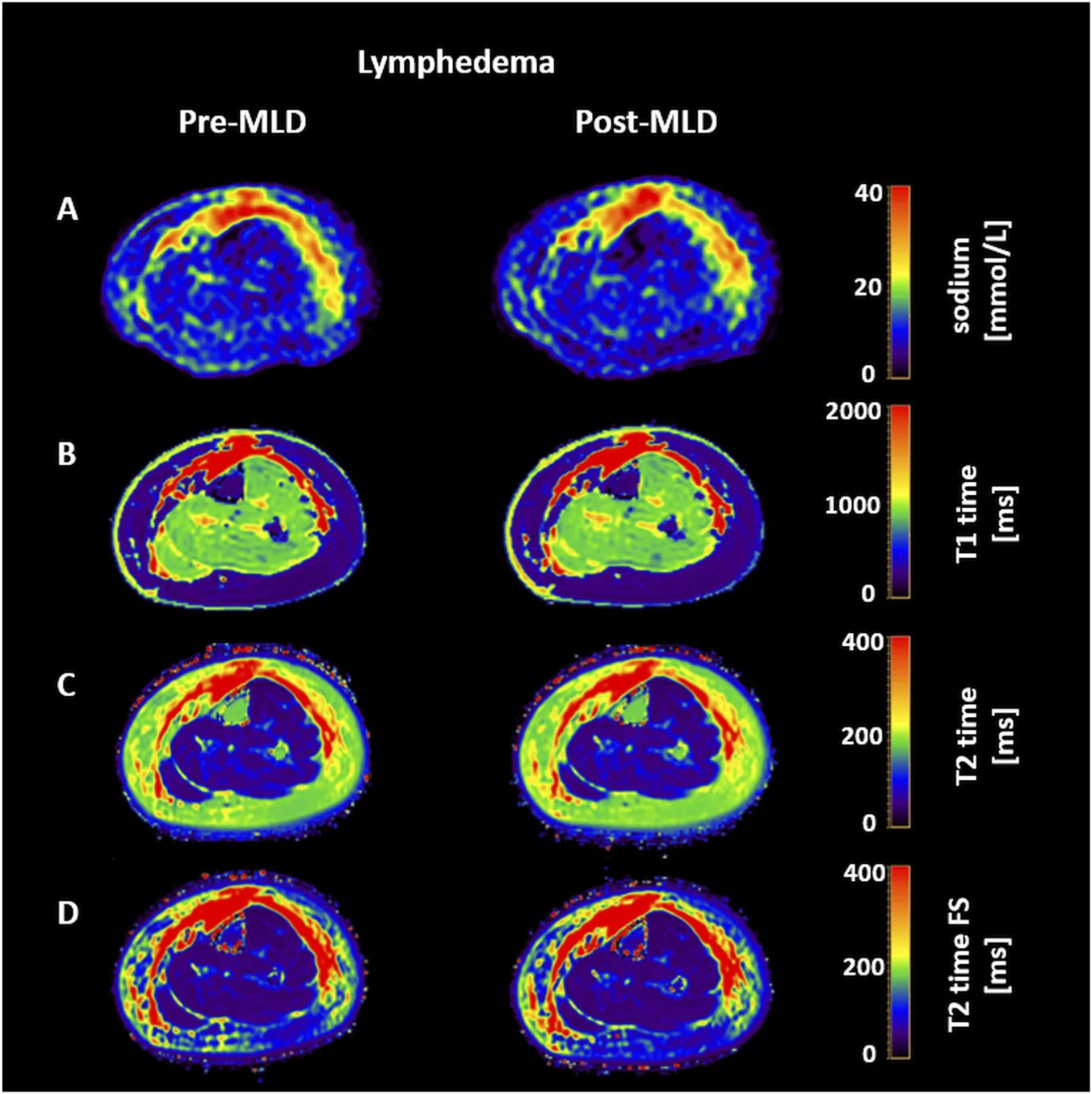
Example MRI scans of a 45-year-old woman with Lymphedema **(A)** Sodium MRI **(B)** T1-mapping **(C)** T2-mapping **(D)** T2-mapping with fat-suppression.

**FIGURE 10:**
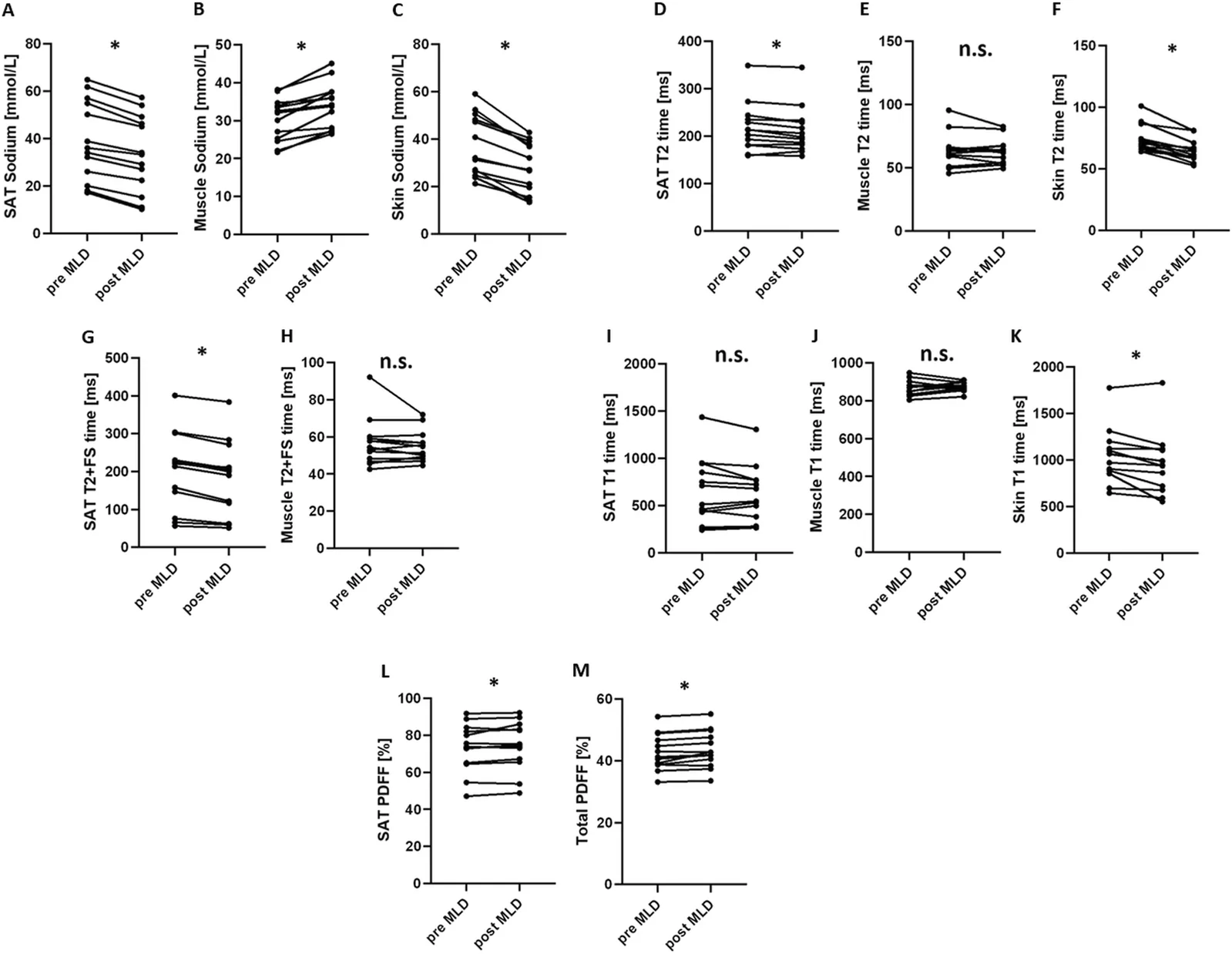
Changes after MLD in Lymphedema. MRI parameters before and after MLD in patients with lymphedema **(A)** SAT sodium **(B)** muscle sodium **(C)** skin sodium **(D)** SAT T2 time **(E)** muscle T2 time **(F)** skin T2 time **(G)** SAT T2 time with fat-suppression **(H)** muscle T2 time with fat-suppression **(I)** SAT T1 time **(J)** muscle T1 time **(K)** skin T1 time (**L**) SAT PDFF **(M)** total PDFF. n. s nonsignificant change, * significant change.

### Baseline-adjusted sensitivity analyses

Baseline-adjusted sensitivity analyses confirmed the main within-group findings. In women with lipedema, reductions in tissue sodium and water-sensitive MRI parameters after MLD remained evident after adjustment for the respective baseline values. Similarly, the lymphedema cohort showed baseline-adjusted changes in the expected direction (Table 3).

## Discussion

The main finding of the present study is that a single session of MLD yields immediate changes in Lipedema tissue composition, as measured by quantitative MRI. Especially tissue sodium levels and water-sensitive MRI parameters decrease (especially T2-times in skin and SAT), suggesting a decrease in sodium-rich interstitial fluid in the examined calf after MLD. These changes are accompanied by a subjective pain relief. Patients with lymphedema served as a positive control group and demonstrated concordant but more pronounced changes in tissue sodium and fluid-sensitive MR parameters after MLD. Ultimately, the study demonstrates that a multiparametric MRI protocol is able to measure post-MLD changes in expected directions.

MLD is commonly employed in conservative treatment of Lipedema as well as in lymphedema (Torres Lacomba et al., 2010). In lymphedema excess interstitial fluid accumulating due to lymphatic insufficiency is mobilized by MLD leading to a reduction in tissue volume after treatment (Brix et al., 2020). A study comparing the effectiveness of MLD and mechanical lymph drainage in 40 patients with secondary lymphedema demonstrated via 3D volumetry that treatment leads to a notable reduction in volume not only following MLD but also mechanical lymph drainage (intermittent pneumatic compression). The volume reduction was most pronounced immediately following the treatment, compared to the 2-day follow-up (Schiltz et al., 2024). An MRI study in breast-cancer related arm lymphedema found that T2 relaxation time was an objective metric that changed after MLD in deep tissue (Donahue et al., 2017). Therefore, as expected, in the present study lymphedema patients demonstrated strong decongestive responses indicated by decreasing sodium levels and shortened water-sensitive parameters in skin and SAT. The small post-MLD increase in muscle sodium observed in lymphedema, in the absence of T2 alterations, most likely indicates a transient shift of interstitial fluid into deeper compartments or along fascial planes rather than genuine muscle edema. T2 mapping in general is sensitive to free water; the drop in skin T2 after MLD and in SAT T2 for lymphedema therefore indicates edema reduction (Donahue et al., 2017). A fat-suppressed T2 (T2-FS) acquisition emphasizes water signals by suppressing fat, increasing sensitivity to small edema shifts (Montant et al., 2015). Additional quantitative MR parameters explored in the present study are Proton Density Fat Fraction (PDFF) and T1 relaxation time mapping. PDFF may rise as water content is reduced (fat constitutes a greater fraction of visible protons) (Schlaeger et al., 2022). T1 mapping is less specific, but may reflect gross changes in tissue composition (Montant et al., 2015; Gaizauskiene et al., 2024; Marty and Carlier, 2019).

In contrast to the treatment of lymphedema, there is an ongoing debate on the presence of soft tissue edema in Lipedema, but MLD is still often reported to be helpful, especially to alleviate pain (Szolnoky et al., 2011; Faerber et al., 2024; Esmer et al., 2020; Herbst et al., 2017; Volkan-Yazıcı et al., 2021; Donahue et al., 2022). However, the treatment mechanism remains poorly understood with some authors suggesting avoiding MLD in Lipedema (Faerber et al., 2024; Gould et al., 2020). Lymphatic insufficiency such as slowed lymphatic flow (Bilancini et al., 1995), enlarged lymphatic vessels (Lohrmann et al., 2009; Brauer and Brauer, 2005) or lymphatic transport abnormalities (Gould et al., 2020) have been reported in Lipedema and may explain to a certain degree the findings at baseline prior to MLD, i.e., increased T1 and T2 relaxation times and elevated sodium levels in subcutaneous adipose tissue and skin in Lipedema patients. Importantly, although prior publications are scarce, the measured sodium values in Lipedema in this study are consistent with previously published data (Crescenzi et al., 2018; Crescenzi et al., 2020). Sodium MRI reflects interstitial sodium accumulation/binding, which may track with edema and/or inflammation. As elevated sodium levels are associated with general inflammatory diseases (Kopp et al., 2013; Jantsch et al., 2015; Kopp et al., 2017), it has been hypothesized that elevated sodium levels in Lipedema may also be linked to inflammation (Crescenzi et al., 2018). The present study corroborates the initial findings of Crescenzi and colleagues (Crescenzi et al., 2018) in a larger cohort with elevated skin and SAT sodium at baseline in Lipedema - correlating with clinical stage and pain (Crescenzi et al., 2018; Crescenzi et al., 2020). Concerning conservative treatment, a previous study evaluated the effects of a 6 week period of physical therapy in women with early-stage Lipedema also including quantitative sodium imaging as an objective parameter of treatment response (Donahue et al., 2022). They found a reduction of sodium levels in skin and SAT with a significant reduction in pain (reported on a visual analog scale). These results are in line with the findings of the present study. However, parameter changes in the study by Donahue et al. were observed over a longer period of several weeks of decongestive therapy making data interpretation difficult due to several possible confounding factors. In the current study we therefore chose to investigate the acute effect of a single MLD-session to rule out other influencing factors. This approach also demonstrated a significant decrease in post-MLD sodium levels in skin and SAT in patients with Lipedema, strengthening the notion that MLD has an immediate effect at a tissue level. Further water-sensitive MRI parameters previously not explored in MLD in Lipedema additionally demonstrate a reduction of tissue water after MLD. Current results therefore suggest that already a single session of MLD leads to a reduction of sodium-rich fluid in the skin and SAT in women with Lipedema.

We observed an immediate reduction in subjective pain intensity levels in women with Lipedema as reported by NRS after MLD corroborating prior clinical observations and pilot trials (Szolnoky et al., 2011; Donahue et al., 2022). The strong correlation between skin sodium and baseline NRS, and the identification of skin sodium as an independent predictor of NRS-values may suggest that interstitial sodium in the dermis may play a role in nociceptive mechanisms in women with Lipedema. Although some patients with lymphedema also reported baseline pain and pain relief following MLD, pain is not a characteristic hallmark of lymphedema. Thus, elevated skin sodium levels alone - which are even more pronounced in lymphedema - do not sufficiently explain the pain experienced in Lipedema. However, in Lipedema, skin sodium demonstrated the strongest associations with both disease stage and pain intensity, underscoring its potential as an objective biomarker candidate for treatment response (Crescenzi et al., 2018; Crescenzi et al., 2020).

This study has several limitations. It was primarily designed and powered for within-group effects, not inter-group comparisons. Although a small healthy control group was included to yield information on normal MRI parameters using the same MRI techniques as in Lipedema and lymphedema patients, it was not possible or intended to match these controls exactly to both groups with Lipedema and lymphedema due to inherent differences between the two patient cohort. However, as far as can be statistically assessed in these small cohorts, healthy controls did not differ significantly regarding age and WtHR distribution. BMI was higher with borderline statistical significance in women with lipedema compared to healthy controls. However, due to the specific distribution and accumulation of subcutaneous fat on the extremities in patients with lipedema, BMI has been shown to be problematic to assess accompanying obesity in these patients. WtHR has been demonstrated to be a more reliable measure for this purpose (Brenner et al., 2023). Importantly, measurements of tissue sodium levels in this small group of healthy volunteers are in agreement with previous published data (Crescenzi et al., 2018; Crescenzi et al., 2020). Group comparisons between patients with lipedema and lymphedema were not performed as the lymphedema cohort was intended as a positive control group to assess whether tissue changes are measurable on MRI in patients with known subcutaneous tissue edema (i.e., lymphedema). A further group comparison is not reasonable as lipedema and lymphedema basically represent different diseases beyond suspected tissue edema in patients with lipedema. As this study was intended as a proof of principle that acute quantifiable changes in Lipedema tissue after MLD are measurable by quantitative MRI, no MLD was performed in the healthy control group. It therefore cannot be ruled out, that changes in MRI-parameters after MLD may also occur in healthy or obese controls as well. In addition, the longer period spent supine during MLD before the second MRI may have contributed to the observed changes in tissue sodium content (Gerhalter et al., 2020). Although participants also rested supine for 10 min before the initial MRI, further studies are necessary to investigate other possible physiological effects on tissue sodium content in more detail. Additionally, sample sizes - especially per Lipedema stage - limit subgroup inference. As this was an exploratory study to generate further research perspectives, no statistical correction for multiple comparisons was performed. All women with lipedema or lymphedema in this study received regular MLD as part of their standard care. As inclusion criteria did not control for the time interval between the last clinical MLD and the study MLD, measured effects may be underestimated in patients who received their last MLD a short period of time before the study examination. On MRI a single axial slice was sampled which may not be representative of more complex changes in the entire leg. Whole-limb imaging approaches may better capture global fluid shifts, but are technically challenging and very time-consuming using current technology. The short window captures acute effects only and durability or cumulative benefits of repeated MLD require further longitudinal studies. However, rather than evaluate these longer-term effects, it was the aim of the present study to investigate tissue changes attributable to MLD alone. In order to avoid possible confounding factors, the study was designed to evaluate the immediate effects of a single MLD session only. Study examinations were undertaken using a 3T (sodium MRI) and 1.5T scanner (relaxometry, fat-quantification, volumetry). This was due to local logistics at our institution. Relaxometry results could therefore not be used to correct for T1/T2 effects on sodium imaging. Future studies should ideally be undertaken at a field strength of 3T for better comparability especially concerning relaxation times.

In conclusion, this quantitative MRI study demonstrates that manual lymphatic drainage has immediate measurable effects on the tissue composition of women with Lipedema. We observed elevated sodium and prolonged T2 relaxation times in the skin and subcutaneous fat of Lipedema legs at baseline, consistent with the presence of sodium-rich interstitial fluid in the tissue. A single session of MLD produced a significant reduction in these MRI indicators of edema, along with prompt pain alleviation. Although the changes were smaller than those seen in lymphedema, tissue sodium content emerged as a particularly important parameter, correlating with Lipedema stage and pain intensity, and decreasing after therapy. Overall, our findings support the clinical use of MLD in Lipedema by providing objective evidence of fluid mobilization and symptom relief. They also encourage further research using advanced imaging to unravel the pathophysiology of Lipedema.

## Data Availability

The original contributions presented in the study are included in the article; further inquiries can be directed to the corresponding author.

## References

[B1] Amato A. C. Amato J. L. Benitti D. (2024). Efficacy of liposuction in the treatment of lipedema: a meta-analysis. Cureus 16 (2), e55260. 10.7759/cureus.55260 38558609 PMC10981502

[B2] Atan T. Bahar-Özdemir Y. (2021). The effects of complete decongestive therapy or intermittent pneumatic compression therapy or exercise only in the treatment of severe lipedema: a randomized controlled trial. Lymphat. Res. Biol. 19 (1), 86–95. 10.1089/lrb.2020.0019 33297826

[B3] Bilancini S. Lucchi M. Tucci S. Eleuteri P. (1995). Functional lymphatic alterations in lipedema. Angiology 46 (4), 333–339. 10.1177/000331979504600408 7726454

[B4] Brauer W. Brauer V. (2005). Altersabhängigkeit des Lymphtransportes beim Lipödem und Lipolymphödem. Lymphologie Forschung Und Praxis 9, 6–9.

[B5] Brenner E. Forner-Cordero I. Faerber G. Rapprich S. Cornely M. (2023). Body-Mass-Index vs. Taille-zu-Größe-Verhältnis bei Patientinnen mit Lipohyperplasia dolorosa (vulgo Lipödem). J. Dtsch. Dermatol Ges. 21 (10), 1179–1187. 10.1111/ddg.15182_g 37845074

[B6] Brix B. Apich G. Roessler A. Ure C. Schmid-Zalaudek K. Hinghofer-Szalkay H. (2020). Fluid shifts induced by physical therapy in lower limb lymphedema patients. J. Clin. Med. 9 (11), 3678. 10.3390/jcm9113678 33207688 PMC7697258

[B7] Cornely M. E. (2014). Fatter through lipids or water: lipohyperplasia dolorosa versus lymphedema. Am. J. Cosmet. Surg. 31, 189–195. 10.5992/AJCS-D-12-00026.1 20871969

[B8] Cornely M. E. (2023). Lipohyperplasia dolorosa: about facts and fiction, clinical presentation and examination. Dermatol. (Heidelb) 74, 566–574. 10.1007/s00105-023-05181-y 37458779

[B9] Crescenzi R. Marton A. Donahue P. M. C. Mahany H. B. Lants S. K. Wang P. (2018). Tissue sodium content is elevated in the skin and subcutaneous adipose tissue in women with lipedema. Obesity 26 (2), 310–317. 10.1002/oby.22090 29280322 PMC5783748

[B10] Crescenzi R. Donahue P. M. C. Petersen K. J. Garza M. Patel N. Lee C. (2020). Upper and lower extremity sodium/fat in lipedema. Obesity 28 (5), 907–915. 10.1002/oby.22778 32270924 PMC7180116

[B11] Dinnendahl R. Tschimmel D. Löw V. Cornely M. Hucho T. (2024). Non-obese lipedema patients show a distinctly altered quantitative sensory testing profile with high diagnostic potential. PAIN Rep. 9, e1155. 10.1097/pr9.0000000000001155 38617100 PMC11013692

[B12] Donahue P. M. C. Crescenzi R. Scott A. O. Braxton V. Desai A. Smith S. A. (2017). Bilateral changes in deep tissue environment after manual lymphatic drainage in BCRL. Lymphat. Res. Biol. 15 (1), 45–56. 10.1089/lrb.2016.0020 28323572 PMC5369395

[B13] Donahue P. M. C. Crescenzi R. Lee C. Garza M. Patel N. Petersen K. J. (2020). MRI and bioimpedance in BCRL. Breast Cancer Res. Treat. 183 (1), 83–94. 10.1007/s10549-020-05765-5 32601969 PMC8086787

[B14] Donahue P. M. C. Crescenzi R. Petersen K. J. Garza M. Patel N. J. Lee C. (2022). Physical therapy in women with early stage lipedema. Lymphat. Res. Biol. 20 (4), 382–390. 10.1089/lrb.2021.0039 34748408 PMC9422785

[B15] Esmer M. Schingale F. J. Unal D. Yazıcı M. V. Güzel N. A. (2020). Physiotherapy and rehabilitation applications in lipedema management: a literature review. Lymphology 53 (2), 88–95. 10.2458/lymph.4659 33190432

[B16] Faerber G. Cornely M. Daubert C. Erbacher G. Fink J. Hirsch T. (2024). S2k guideline lipedema. J. Dtsch. Dermatol Ges. 22 (9), 1303–1315. 10.1111/ddg.15513 39188170

[B17] Gaizauskiene K. Leketaite K. Glaveckaite S. Valeviciene N. (2024). CMR T1/T2 mapping in acute myocarditis: systematic review. Medicina 60 (7), 1162. 10.3390/medicina60071162 39064591 PMC11279077

[B18] Gerhalter T. Gast L. V. Marty B. Uder M. Carlier P. G. Nagel A. M. (2020). Assessing the variability of 23Na MRI in skeletal muscle tissue: reproducibility and repeatability of tissue sodium concentration measurements in the lower leg at 3 T. NMR Biomed. 33, e4279. 10.1002/nbm.4279 32125054

[B19] Gould D. J. El-Sabawi B. Goel P. Badash I. Colletti P. Patel K. M. (2020). Lymphatic transport abnormalities in primary lipedema. J. Reconstr. Microsurg 36 (2), 136–141. 10.1055/s-0039-1697904 31546262

[B20] Herbst K. L. Ussery C. Eekema A. (2017). Pilot study: whole body manual subcutaneous adipose tissue (SAT) therapy improved pain and SAT structure in women with lipedema. Horm. Mol. Biol. Clin. Investig. 33 (2), 20170035. 10.1515/hmbci-2017-0035 28930626

[B21] Jantsch J. Schatz V. Friedrich D. Schröder A. Kopp C. Siegert I. (2015). Cutaneous Na^+^ storage strengthens antimicrobial barrier function. Cell Metab. 21 (3), 493–501. 10.1016/j.cmet.2015.02.003 25738463 PMC4350016

[B22] Kopp C. Linz P. Dahlmann A. Hammon M. Jantsch J. Mülle D. N. (2013). 23Na MRI-Determined tissue sodium in health/hypertension. Hypertension 61 (3), 635–640. 10.1161/HYPERTENSIONAHA.111.00566 23339169

[B23] Kopp C. Beyer C. Linz P. Dahlmann A. Hammon M. Jantsch J. (2017). Na^+^ deposition in systemic sclerosis skin by 23Na MRI. Rheumatology 56 (4), 556–560. 10.1093/rheumatology/kew371 28013199 PMC6482592

[B24] Kruppa P. Georgiou I. Biermann N. Prantl L. Klein-Weigel P. Ghods M. (2020). Lipedema – pathogenesis, diagnosis, and treatment options. Dtsch. Arztebl Int. 117 (22-23), 396–403. 10.3238/arztebl.2020.0396 32762835 PMC7465366

[B25] Kruppa P. Crescenzi R. Faerber G. Forner-Cordero I. Cornely M. Shayan R. (2026). Lipedema world alliance Delphi consensus-based position paper on the definition and management of lipedema: results from the 2023 lipedema world Congress in potsdam. Nat. Commun. 17 (1), 427. 10.1038/s41467-025-68232-z 41519859 PMC12796449

[B26] Lohrmann C. Foeldi E. Langer M. (2009). MR imaging of the lymphatic system in lipedema/lipo-lymphedema. Microvasc. Res. 77 (3), 335–339. 10.1016/j.mvr.2009.01.005 19323976

[B27] Marty B. Carlier P. G. (2019). Physiological and pathological skeletal muscle T1 changes quantified using a fast inversion-recovery radial NMR imaging sequence. Sci. Rep. 9 (1), 6852. 10.1038/s41598-019-43398-x 31048765 PMC6497638

[B28] Montant P. Sigovan M. Revel D. Douek P. (2015). MR imaging assessment of myocardial edema with T2 mapping. Diagn Interv. Imaging 96 (9), 885–890. 10.1016/j.diii.2014.07.008 25697831

[B29] Nankam P. A. N. Cornely M. Klöting N. Blüher M. (2022). Is subcutaneous adipose tissue expansion in people living with lipedema healthier and reflected by circulating parameters? Front. Endocrinol. 13, 1000094. 10.3389/fendo.2022.1000094 36387874 PMC9659629

[B30] Nowak S. Henkel A. Theis M. Luetkens J. Geiger S. Sprinkart A. M. (2023). Deep learning for standardized, MRI-Based quantification of subcutaneous and subfascial tissue volume for patients with lipedema and lymphedema. Eur. Radiol. 33 (2), 884–892. 10.1007/s00330-022-09047-0 35976393 PMC9889496

[B31] Schiltz D. Eibl D. Mueller K. Biermann N. Prantl L. Taeger C. D. (2024). Therapist versus Machine—immediate effects of manual versus mechanical lymphatic drainage in patients with secondary Lymphedema. J. Clin. Med. 13 (5), 1277. 10.3390/jcm13051277 38592094 PMC10931544

[B32] Schlaeger S. Weidlich D. Zoffl A. Becherucci E. A. Kottmaier E. Montagnese F. (2022). Voxel-based analysis of water T2 in the presence of fatty infiltration. NMR Biomed. 35 (12), e4805. 10.1002/nbm.4805 35892264

[B33] Szolnoky G. Varga E. Varga M. Tuczai M. Dósa-Rácz E. Kemény L. (2011). Lymphedema treatment decreases pain intensity in lipedema. Lymphology 44 (4), 178–182. 22458119

[B34] Torres Lacomba M. Yuste Sánchez M. J. Zapico Goñi A. Prieto Merino D. Mayoral del Moral O. Cerezo Téllez E. (2010). Early physiotherapy to prevent lymphoedema after breast cancer surgery. BMJ 340, b5396. 10.1136/bmj.b5396 20068255 PMC2806631

[B35] Volkan-Yazıcı M. Yazici G. Esmer M. (2021). The effects of complex decongestive physiotherapy applications on lower extremity circumference and volume in patients with lipedema. Lymphat. Res. Biol. 19 (1), 111–114. 10.1089/lrb.2020.0080 33124947

[B36] Vyas A. Adnan G. (2024). in Statpearls Lipedema (Treasure Island, FL: StatPearls Publishing). Available online at: http://www.ncbi.nlm.nih.gov/books/NBK573066/ (Accessed August 20, 2024).

